# The Nociceptin Receptor (NOP) Agonist AT-312 Blocks Acquisition of Morphine- and Cocaine-Induced Conditioned Place Preference in Mice

**DOI:** 10.3389/fpsyt.2018.00638

**Published:** 2018-11-29

**Authors:** Nurulain T. Zaveri, Paul V. Marquez, Michael E. Meyer, Abdul Hamid, Kabirullah Lutfy

**Affiliations:** ^1^Astraea Therapeutics, LLC, Mountain View, California, CA, United States; ^2^Department of Pharmaceutical Sciences, College of Pharmacy, Western University of Health Sciences, Pomona, CA, United States

**Keywords:** morphine, cocaine, conditioned place preference, AT-312, NOP agonist, NOP receptor knockout, polydrug addiction, addiction pharmacotherapy

## Abstract

Treatment of drug addiction remains an unmet medical need due to the dearth of approved pharmacotherapies. There are no approved treatments for cocaine addiction, whereas the current opioid crisis has revealed the stark reality of the limited options to treat prescription and illicit opioid abuse. Preclinical studies in rodents and nonhuman primates have shown that orphanin FQ/nociceptin (N/OFQ), the endogenous ligand for the nociceptin opioid receptor (NOP) reduces the rewarding effects of several abused substances, including opioids, psychostimulants and alcohol. A few nonpeptide small-molecule NOP agonists have also shown efficacy in attenuating the rewarding effects of various abused drugs. We previously demonstrated that a high affinity small-molecule NOP agonist AT-312 selectively reduced the rewarding effects of ethanol in the conditioned place preference paradigm in mice. In the present study, we examined if AT-312 (3 mg/kg, i.p. or s.c. respectively), would alter the rewarding action of morphine (7.5 mg/kg, s.c.) or cocaine (15 mg/kg, i.p.). The effect of AT-312 on morphine- and cocaine-induced motor stimulation was also assessed on the conditioning days. The role of the NOP receptor in the effects of AT-312 was further confirmed by conducting the place conditioning experiments in NOP knockout mice and compared to their wild-type controls. Our results showed that AT-312 significantly reduced the acquisition of morphine and cocaine CPP in wild-type mice but not in mice lacking NOP receptors. AT-312 also suppressed morphine-induced and completely abolished cocaine-induced motor stimulation in NOP wild-type mice, but not in NOP knockout mice. These results show that small-molecule NOP receptor agonists have promising efficacy for attenuating the rewarding effects of morphine and cocaine, and may have potential as pharmacotherapy for opioid and psychostimulant addiction or for treating polydrug addiction.

## Introduction

Addiction pharmacotherapy remains an area severely in need of new approaches and new targets, given that there are no approved therapies for cocaine addiction and the limited suboptimal options available for those addicted to opioids fueling the opioid crises. Addiction to more than one drug is also quite prevalent, but there are few, if any, appropriate pharmacotherapies that can address polydrug addiction effectively. Most addictive substances cause an increase in dopamine release in the mesolimbic areas of the reward circuitry, albeit through different mechanisms. Cocaine blocks the dopamine transporter and increases dopamine levels in the nucleus accumbens (NAc) (1–3); whereas opioid drugs increase dopamine levels in the VTA and NAc through their action at the mu opioid receptor (4, 5). Even the endogenous opioid peptides, beta-endorphin and enkephalins increase the activity of the mesolimbic dopaminergic neurons and stimulate dopamine release in the NAc (6). In fact, the endogenous opioid system has been shown to be involved in the rewarding actions of other drugs of abuse such as cocaine and alcohol (7–9). Therefore, approaches that inhibit the dopaminergic transmission in the mesolimbic circuitry may be useful for reducing rewarding effects of many different addictive drugs.

The endogenous peptide, nociceptin/orphanin FQ (N/OFQ), acting through the nociceptin opioid peptide (NOP) receptor, is present in several areas of the brain associated with reward and stress pathways such as the ventral tegmental area, prefrontal cortex, amygdala and lateral hypothalamus (10, 11). The NOP receptor and its endogenous ligand N/OFQ are the fourth members of the opioid family of G protein-coupled receptors mu, delta and kappa and their endogenous ligands endorphins, enkephalins and dynorphin (12, 13). Unlike the classical opioid ligands, however, N/OFQ has a broad inhibitory effect on multiple neurotransmitter systems involved in drug reward and has been shown to decrease drug-induced dopamine levels in the nucleus accumbens (14, 15). Intracerebroventricular (i.c.v.) administration of N/OFQ has been shown to block morphine-induced dopamine release in the NAc (14, 16–19). We and others have shown that central administration of the N/OFQ peptide decreases cocaine-induced dopamine release in the NAc in rats (20, 21). Consistent with these observations, i.c.v. administration of N/OFQ blocks the rewarding effects of morphine, cocaine and alcohol in animal models of drug reward such as the conditioned place preference (CPP) (17, 22–24). Targeting the NOP-N/OFQ system is therefore a potential approach to reduce the rewarding effects of multiple abused substances and develop pharmacotherapy to treat addiction to various drugs and possibly polydrug addiction (25–28).

Some effort along these lines has been expended with few synthetic small-molecule NOP agonists, producing equivocal results. The nonpeptide small-molecule NOP agonist Ro 64-6198 was reported to block acquisition and reinstatement of morphine-induced CPP after intraperitoneal (i.p.) administration (29), and the expression, acquisition and reinstatement of alcohol-induced CPP in mice (24). However, i.p. Ro 64-6198 was also reported to produce a place preference in rats through a purported dopaminergic mechanism, and not through an opioid or NOP-related mechanism (30). A different NOP agonist Ro 65-6570 on the other hand, blocked opioid- and cocaine-induced CPP in rats in the same study, and this effect was reversed by a NOP antagonist J-113397, confirming the role of the NOP receptor in the anti-rewarding actions of Ro 65-6570 (30). NOP agonist SCH221510 was shown to reduce opioid (remifentanil) self-administration in rats only when administered intracisternally but not systemically (31). We recently reported that SCH221510 showed a modest inhibition of ethanol-induced CPP in mice at high doses given i.p. (32). On the other hand, in the same study, we showed that a novel and selective NOP agonist AT-312 showed a significant and robust inhibition of ethanol CPP in mice at doses lower than that of SCH221510. Moreover, this effect was absent in mice lacking the NOP receptor, confirming the NOP-targeted inhibition of ethanol CPP by AT-312 (32).

The aims of this study were to determine the efficacy of AT-312 in attenuating the rewarding effects of other drugs of abuse such as morphine and cocaine. To confirm the pharmacological mechanism of the effect of AT-312, we conducted the CPP experiments in mice lacking the NOP receptor and their wild-type littermates. In addition, we also characterized the effect of AT-312 on the locomotor stimulation produced by morphine or cocaine in wild-type and NOP knockout mice.

## Materials and methods

### Subjects

Mice lacking the NOP receptor and their wild-type controls, fully backcrossed on a C57BL/6J mouse strain, were bred in house, and weaned at the age of 21–24 days, prior to genotyping. Mice between the ages of 2–4 months were used for these experiments. Mice were housed 2–4 per cage with free access to laboratory chow and water and maintained under a 12 h light/ 12 h dark cycle. All experimental procedures were approved by the Institutional Animal Care and Use Committee at Western University of Health Sciences (Pomona, CA) and were in accord with the NIH Guide for the Use and Care of Animal in Research.

### Drugs

AT-312 ((1-(1-((cis)-4-isopropylcyclohexyl)piperidin-4-yl)-1H-indol-2-yl)methanol) was synthesized at Astraea Therapeutics. The details of the synthesis and the *in vitro* pharmacological profile of AT-312 at the opioid receptors have been previously reported (32). AT-312 was dissolved in 1–2% DMSO and then diluted to the desired concentration with 0.5% aqueous hydroxypropylcellulose (HPC) and injected subcutaneously (s.c.) or intraperitoneally (i.p.) at a dose of 3 mg/kg in a volume of 0.1 ml/10 g of body weight. The dose of AT-312 was selected based on our previous study (32). Controls received 0.1 ml/10 g of body weight of the appropriate vehicle (1–2% DMSO in 0.5% of HPC). Morphine sulfate and cocaine hydrochloride were obtained from NIDA Drug Supply and dissolved in normal saline. The doses of morphine and cocaine are as their salt forms.

### Experimental procedures

#### Effect of AT-312 on morphine-induced CPP in wild-type and NOP knockout mice

A detailed description of the CPP apparatus and procedure is given elsewhere (33). Briefly, female mice lacking NOP receptor and their wild-type controls were tested for baseline place preference on day 1, in which each mouse was placed in the neutral central chamber and allowed to freely explore the CPP chambers for 15 min. The amount of time that mice spent in each conditioning chamber was recorded. There was no initial preference for any of the CPP chambers, i.e., the CPP paradigm was unbiased. The following day, mice were treated with either vehicle or AT-312 (3 mg/kg, i.p.) followed, 5 min later, by morphine or saline (morphine vehicle) (7.5 mg/kg, s.c.) and confined to the vehicle-paired chamber (VPCh; if they received vehicle and then saline) or drug-paired chamber (DPCh; if they received vehicle or AT-312 before morphine) for 60 min. The dose of AT-312 and morphine, the conditioning time, and the time of AT-312 administration with respect to morphine administration were based on our previous studies (32, 34). The CPP procedure was carried out in a counterbalanced manner, in which some animals were treated with vehicle or AT-312 followed by morphine in the morning and some in the afternoon. If they received vehicle or AT-312 followed by morphine in the morning, they received vehicle followed by saline in the afternoon on that day. If they received vehicle followed by saline in the morning, they received conditioning with vehicle or AT-312 followed by morphine in the afternoon. Locomotor activity, measured as distance traveled, was also recorded on each conditioning day. The twice daily conditioning continued for three consecutive days on days 2–4. Mice were then tested for post conditioning place preference on day 5 in a drug-free state, as described for day 1.

#### Effect of AT-312 on cocaine-induced CPP in wild-type and NOP knockout mice

We also assessed the effect of AT-312 on cocaine-induced CPP in mice. The CPP procedure was the same as described above for morphine except that male mice were used for this experiment. On conditioning days, mice were treated with vehicle or AT-312 (3 mg/kg, s.c.) and 5 min later with cocaine (15 mg/kg, i.p.), and then confined to the DPCh for 30 min. Other mice received vehicle and 5 min later saline and were confined to the VPCh for 30 min. In the afternoon, mice received the alternative treatment (e.g., vehicle followed by saline; if they were treated with vehicle or AT-312 followed by cocaine) and were confined to the opposite conditioning chamber (e.g., chamber with the rod floor if mice received the other treatment in the chamber with mesh floor) for 30 min. This twice-daily conditioning lasted for 3 consecutive days and mice were tested for post conditioning place preference on day 5, as described above.

#### Data analysis

Values represent mean (± S.E.M.) of the amount of time (sec) that mice spent in the CPP chambers on the preconditioning (day 1, D1) and postconditioning (day 5, D5) test days or the distance traveled (cm) during the conditioning days. Data were analyzed using three-way repeated measures analysis of variance (ANOVA) followed by the *post-hoc* Tukey's test. The factors were pretreatment (i.e., vehicle vs. AT-312) and treatment (saline vs. cocaine) and time (day 1 and day 5). A *P* < 0.05 was considered significant.

## Results

### AT-312 reduced CPP induced by morphine in wild-type but not NOP knockout mice

**Figure 1:**
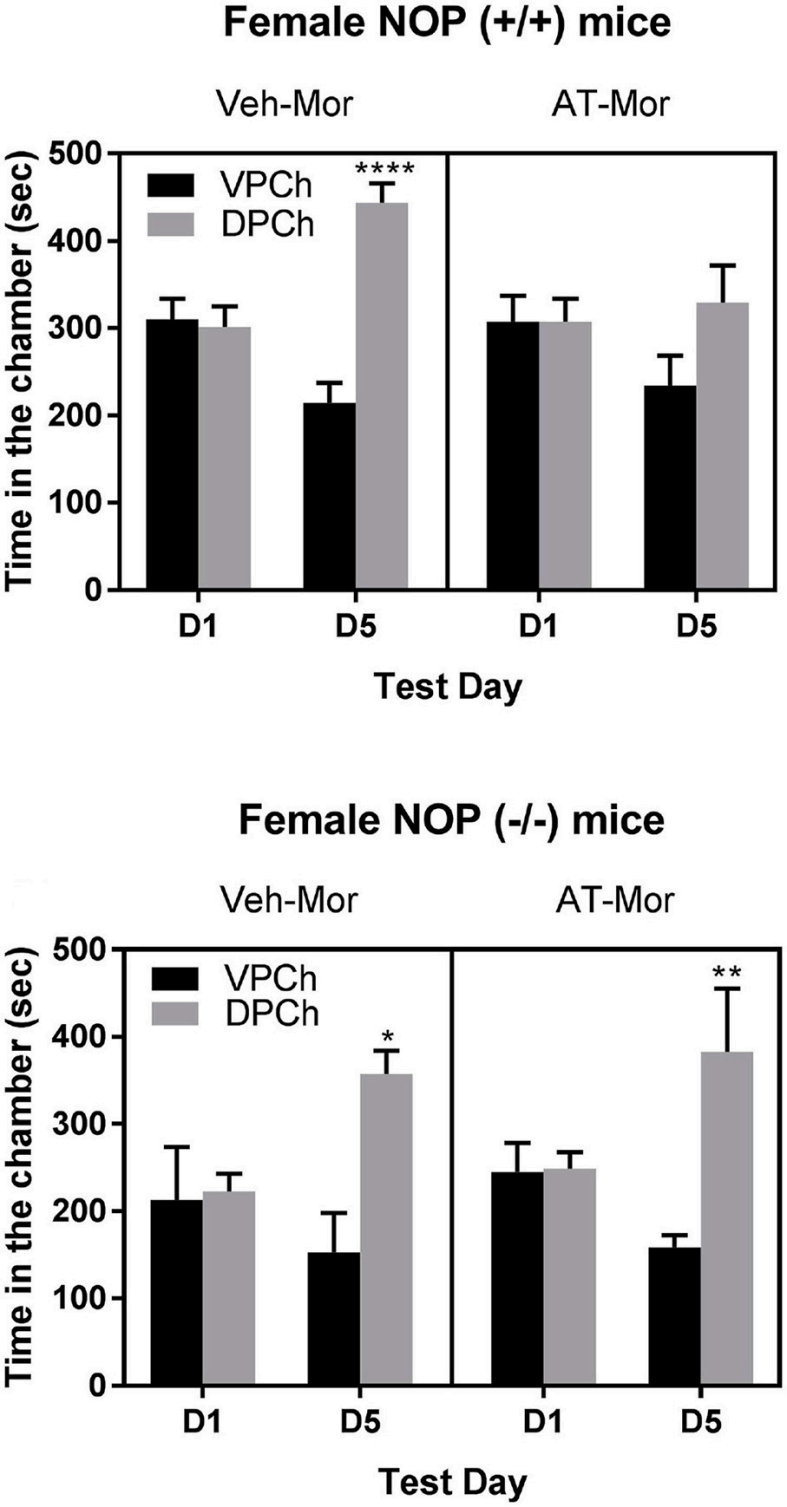
Effects of AT-312 on morphine-induced CPP in mice lacking NOP and their wild-type littermates/controls. Mice (*n* = 6 mice per treatment for each genotype) were tested for baseline place preference on day 1, conditioned with morphine (7.5 mg/kg, s.c.) in the presence and absence of AT-312 (3 mg/kg, i.p.) on days 2–4 and then tested for CPP on day 5. Vehicle or AT-312 was given 5 min before morphine on each conditioning day to wild-type **(Upper)** and knockout **(Lower)** mice. *P < 0.05; **P < 0.01; ****P < 0.0001, a significant increase in the amount of time that mice spent in the drug-paired chamber (DPCh) compared to vehicle-paired chamber (VPCh) on this day.

Figure 1 shows the amount of time (sec) that mice lacking NOP and their wild-type controls spent in the conditioning chambers before (day 1, D1) and after (day 5, D5) conditioning. A three-way ANOVA of the data in wild-type mice showed a significant effect of treatment [*F*_1, 40_ = 14.88; *P* < 0.001] and a significant interaction between time and treatment [*F*_1, 40_ = 16.61; *P* < 0.001] and a trend toward an interaction between time, pretreatment and treatment [*F*_1, 40_ = 3.09; *P* < 0.09]. The Tukey's *post-hoc* test revealed that morphine induced a robust CPP response in vehicle-treated control mice, as evidenced by a significant (*P* < 0.0001) increase in the amount of time that mice spent in the drug-paired chamber (DPCh) compared to vehicle-paired chamber (VPCh) on the postconditioning test day (D5; Figure 1; upper panel; compare DPCh vs. VPCh for the Veh-Mor group). However, this response was abolished in mice treated with AT-312 in conjunction with morphine on each conditioning day (Figure 1, upper panel; compare DPCh vs. VPCh for the AT-Mor group). In contrast, AT-312 did not alter morphine-induced CPP in mice lacking NOP (Figure 1, lower panel). The Tukey *post-hoc* test revealed a significant increase in the amount of time that mice of each pretreatment group (vehicle or AT-312 pretreated) spent in the DPCh vs. VPCh on day 5 (*P* < 0.05). These results indicate that AT-312 blocked the acquisition of morphine-induced CPP via the NOP receptor.

### AT-312 reduced morphine-induced motor stimulation during conditioning in wild-type but not in NOP knockout mice

**Figure 2:**
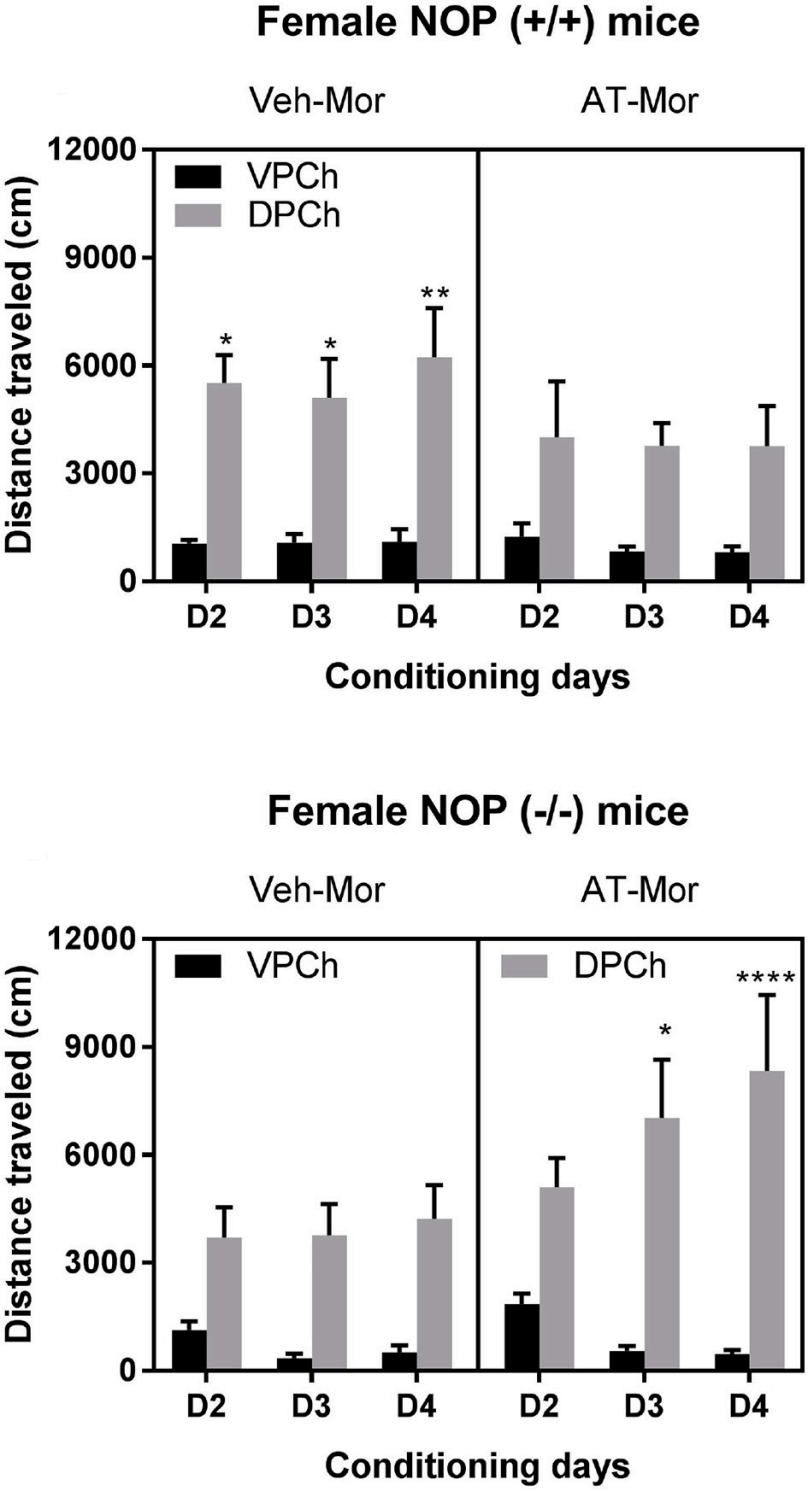
Effects of AT-312 on morphine-induced motor stimulation during the conditioning days in the CPP chambers in mice lacking NOP and their wild-type littermates/controls. On each conditioning day, wild-type **(Upper)** and NOP knockout **(Lower)** mice were treated with vehicle or AT-312 (3 mg/kg; i.p.; *n* = 6 mice per treatment for each genotype) 5 min before morphine (7.5 mg/kg, s.c.) and distance traveled by mice in the morphine-paired chamber (DPCh) was recorded for 60 min each day. Distance traveled by the mice in the saline-paired chamber (VPCh) was also recorded, in which mice were injected with vehicle and 5 min later with saline. *P < 0.05, **P < 0.01 and ****P < 0.0001, a significant increase in distance traveled by mice in the DPCh vs. their respective VPCh on this day.

We found that AT-312 reduced the motor stimulatory effect of morphine in wild-type mice (Figure 2, upper right panel) but not in mice lacking NOP receptor (Figure 2, lower right panel). Three-way ANOVA of the data in wild-type mice revealed a significant effect of pretreatment [*F*_(1, 60)_ = 4.03, *P* < 0.05] and a significant effect of treatment [*F*_(1, 60)_ = 62.11, *P* < 0.0001] but no significant interaction between pretreatment, treatment and time [*F*_(2, 60)_ = 0.11, *P* > 0.05]. The Tukey's *post-hoc* test revealed that vehicle-pretreated mice traveled significantly greater distances in the chamber conditioned with morphine compared to saline on each conditioning day (*P* < 0.05; Figure 2, upper left panel). However, this response was attenuated in wild-type mice pretreated with AT-312 as evidenced by no significant difference in distance traveled after morphine treatment compared to saline in this group (*P* > 0.05; Figure 2, upper right panel). On the other hand, the *post-hoc* test showed that mice lacking NOP receptors conditioned with morphine traveled significantly more distance compared to saline-conditioned animals on each conditioning day (Figure 2, lower left panel). However, this response was not reduced in NOP knockout mice treated with AT-312 prior to morphine (Figure 2, lower right panel). Interestingly, AT-312 not only did not reduce morphine-induced motor stimulation in the NOP knockout mice but it appeared that the stimulatory action of morphine was increased in these mice in the presence of AT-312. (Figure 2, lower right panel, compare day 2 vs. day 4) However, this response was not different than that in the Veh-Mor knockout mice (*P* > 0.05). Overall, these results suggest the involvement of the NOP receptors in the suppressive effect of AT-312 on the motor stimulation produced by morphine.

### AT-312 reduced acquisition of cocaine CPP in wild-type but not NOP knockout mice

**Figure 3:**
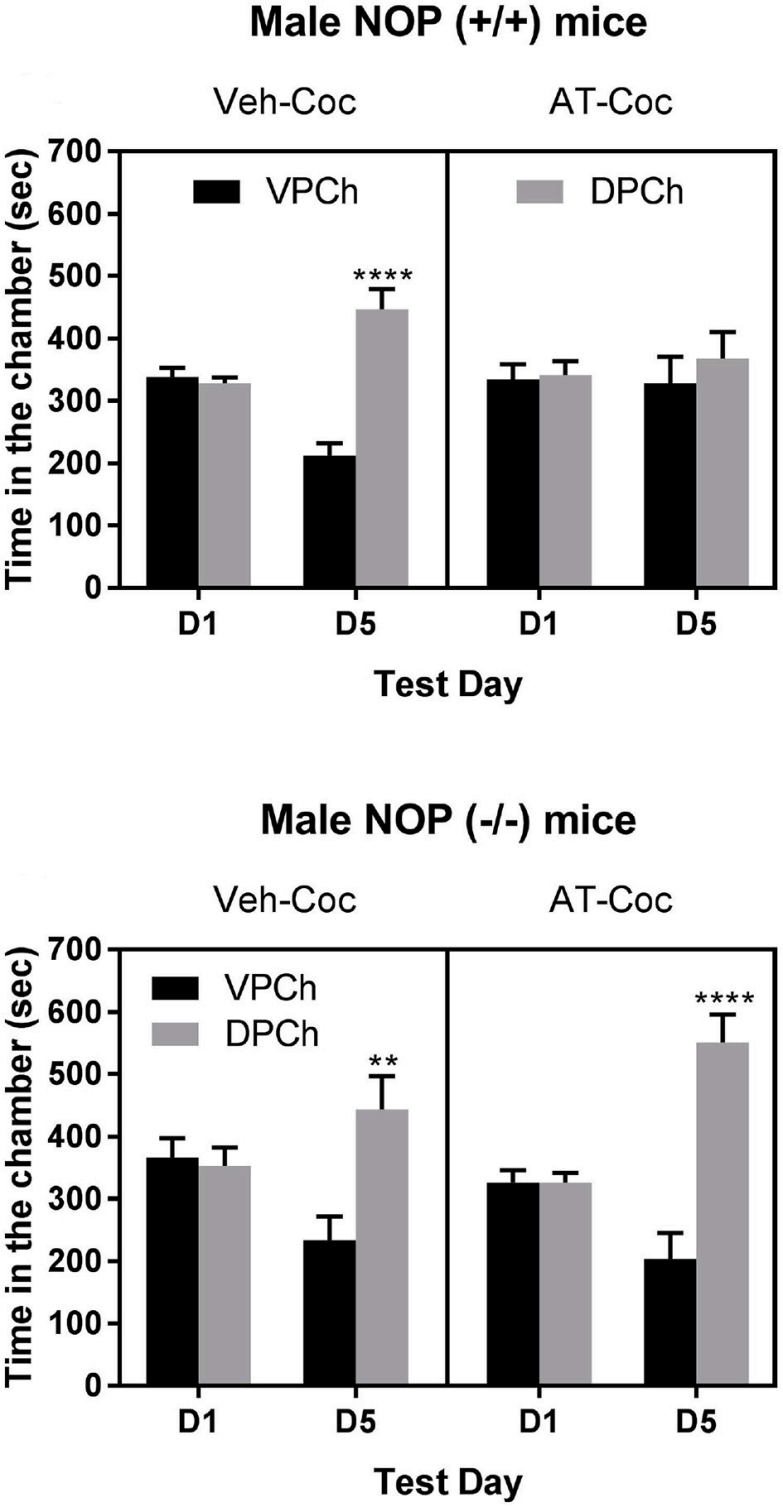
Effect of AT-312 on cocaine-induced CPP in wild-type (**Upper**; *n* = 13 mice per treatment) or knockout (**Lower**; *n* = 10 mice per treatment) mice. Data are mean (±SEM) of the amount of time that mice spent in the vehicle-paired chamber (VPCh) and drug-paired chamber (DPCh) on the preconditioning (D1) and postconditioning (D5) test days. **P < 0.01; ****P < 0.0001, a significant increase in the amount of time that mice spent in the DPCh vs. its respective VPCh on D5.

Figure 3 shows the amount of time that wild-type (upper panel) and knockout (lower panel) mice spent in the vehicle-paired chamber (VPCh) and drug-paired chamber (DPCh) on the preconditioning (D1) and postconditioning (D5) test days. Three-way repeated measures ANOVA of the data in wild-type mice showed a significant effect of treatment [*F*_(1, 96)_ = 11.35; *P* < 0.001], a significant effect of pretreatment × treatment interaction [*F*_(1, 96)_ = 4.96: *P* < 0.03] and a significant interaction between time × pretreatment × treatment [*F*_(1, 96)_ = 6.93; *P* < 0.01]. The Tukey's *post-hoc* test showed that cocaine induced a robust CPP in vehicle-treated control, as evidenced by a significant increase in the amount of time that mice spent in the DPCh compared to VPCh on the postconditioning day (Figure 3, upper left panel; compare DPCh vs. VPCh on day 5 for the Veh-Coc group). In contrast, this response was abolished in mice treated with AT-312 prior to cocaine on each conditioning day (Figure 3, upper right panel; compare DPCh vs. VPCh on day 5 in the AT-Coc group). On the other hand, AT-312 had no effect on the place preference induced by cocaine in mice lacking NOP (Figure 3, lower left panel). Three-way ANOVA showed a significant effect of time × treatment interaction [*F*_1, 40_ = 12. 62, *P* < 0.001] but no significant interaction between pretreatment × treatment [*F*_1, 40_ = 0.01; *P* > 0.05] and no significant interaction between pretreatment × treatment and time [*F*_1, 40_ = 0.05; *P* > 0.05]. The Tukey's *post-hoc* test revealed a significant increase in the amount of time that mice spent in DPCh compared to VPCh on the postconditioning day regardless of the pretreatment (*P* < 0.05). Overall, these results indicate that AT-312 blocked cocaine-induced CPP via the NOP receptor.

**Figure 4:**
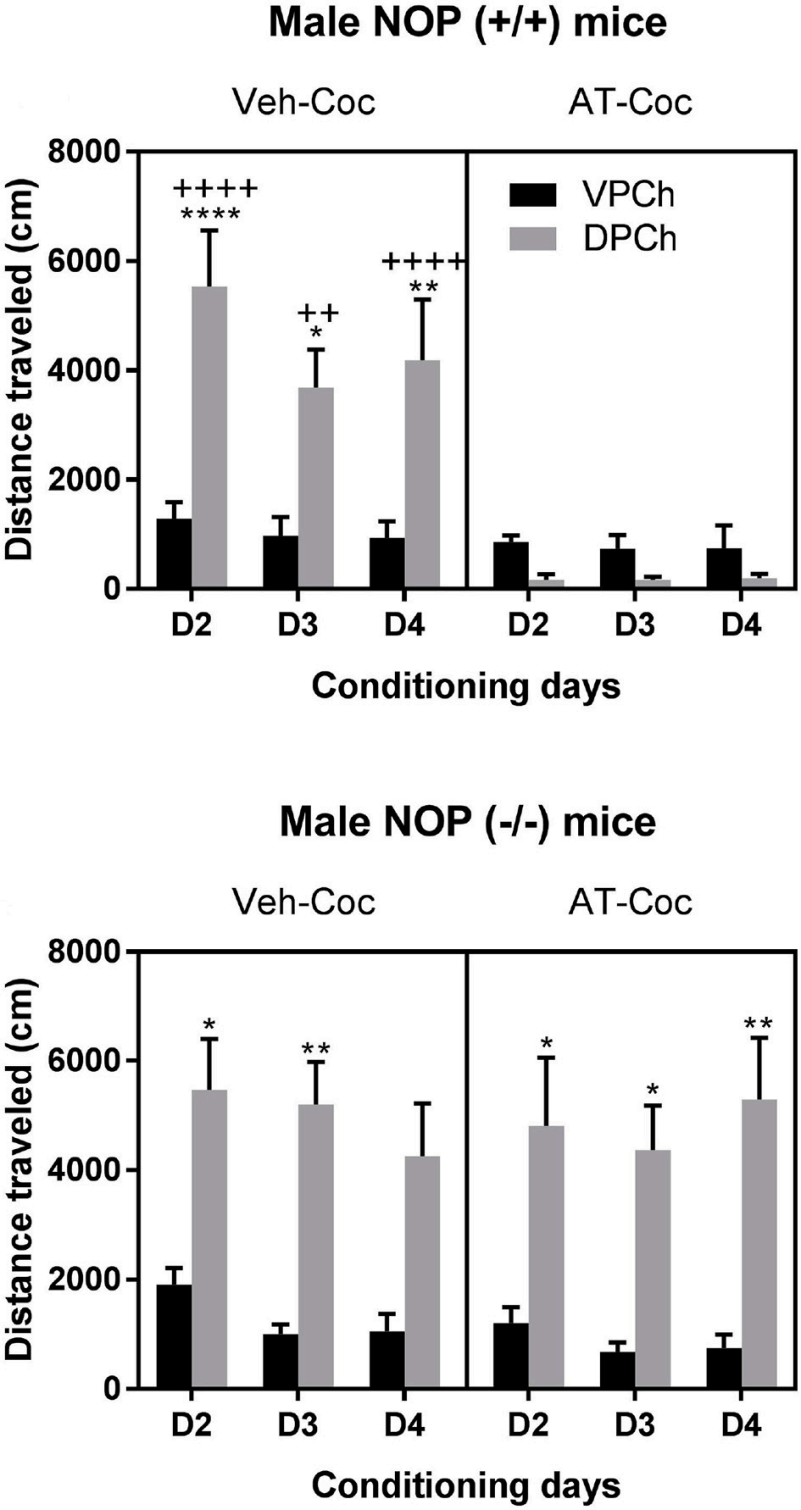
Effects of AT-312 on cocaine-induced motor stimulation during conditioning in wild-type and knockout mice. Motor activity was measured in a subset of animals (*n* = 6 mice per genotype for each treatment) and the experiment was repeated three times (*n* = 2 mice of each genotype per treatment for each cohort). On each conditioning day, wild-type **(Upper)** and NOP knockout **(Lower)** mice were treated with vehicle or AT-312 (3 mg/kg) 5 min before cocaine (15 mg/kg, i.p.) and distance traveled by mice in the drug-paired chamber (DPCh) was recorded for 30 min each day. The distance traveled by mice in the saline-paired chamber (VPCh) was also recorded, in which mice were injected with vehicle and 5 min later with saline. *P < 0.05, **P < 0.01 and ****P < 0.0001, a significant increase in distance traveled by mice in the DPCh vs. their respective VPCh on this day. ^++^*P* < 0.01 and ^++++^*P* < 0.0001 vs. DPCh in mice treated with AT-312 followed by cocaine on this day.

Figure 4 shows the effect of AT-312 on the motor stimulatory action of cocaine in wild-type (upper panel) and NOP knockout (lower panel) mice. Three-way ANOVA revealed a significant interaction between pretreatment and treatment [*F*_(1, 60)_ = 43.9, *P* < 0.0001] but no significant time × treatment [*F*_(2, 60)_ = 0.63; *P* > 0.05] or time × pretreatment × treatment interaction [*F*_(2, 60)_ = 0.65; *P* > 0.05]. *Post-hoc* analyses of the data showed that cocaine increases motor activity compared to saline in wild-type mice (Figure 4, upper left panel; compare distance traveled between DPCh and VPCh in mice pretreated with vehicle and conditioned with cocaine, i.e., Veh-Coc group). This response was completely blocked in wild-type mice pretreated with AT-312 (Figure 4, upper right panel; compare distance traveled in the DPCh vs. VPCh in mice AT-Coc group). In contrast, AT-312 failed to alter the motor stimulatory action of cocaine in mice lacking NOP (Figure 4, lower right panel). Three-way ANOVA revealed a significant effect of treatment [*F*_(1, 60)_ = 82.02; *P* < 0.0001] but no significant interaction between pretreatment and treatment [*F*_(1, 60)_ = 0.12; *P* > 0.05] or time × pretreatment × treatment interaction [*F*_(2, 60)_ = 0.43; *P* > 0.05]. The *post-hoc* test showed a significant increase in distance traveled by cocaine in mice lacking NOP regardless of the pretreatment (Figure 4, lower panel). Together, these results suggest that AT-312 abolished the motor stimulatory action of cocaine in wild-type but not knockout mice.

## Discussion

The main findings of the present study are (i) AT-312 significantly attenuates acquisition of CPP to morphine and cocaine in the CPP paradigm; (ii) AT-312 attenuates morphine and cocaine CPP through its action at the NOP receptor, as this attenuation is absent in mice lacking the NOP receptor; and (iii) AT-312 blocks morphine- and cocaine-induced locomotor stimulation.

Previous studies have shown that N/OFQ reduces the rewarding action of morphine (17, 35), raising the possibility that nonpeptide NOP agonists may reduce the rewarding effects of opioids. Indeed, the selective small-molecule NOP agonist Ro 64-6198 blocked the acquisition and reinstatement of morphine CPP in mice (29). A chemically-related NOP agonist Ro 65-6570 demonstrated significant “anti-opiate” effects in rats by reducing CPP of a variety of opiate drugs including morphine, oxycodone, and heroin, particularly when administered within a 15-min (but not 30-min) pretreatment interval. We previously reported that another chemically distinct, and modestly selective NOP agonist AT-202 suppressed the acquisition of morphine CPP in mice, and this effect was reversed by pretreatment with a selective NOP antagonist SB-612111 (36), confirming the NOP receptor as the target for the anti-rewarding effect of AT-202. Results in this study showed that morphine induced a robust acquisition of CPP response in NOP wild-type mice, and this response was significantly reduced in mice pretreated with AT-312 in conjunction with morphine. The inhibitory effect of AT-312 on acquisition of morphine CPP appears to be consistent with that observed with other previously reported NOP agonists. Morphine also showed a robust CPP in mice lacking the NOP receptor; however, pretreatment with AT-312 had no effect on morphine place preference in these mice. This lack of an effect in the NOP knockout mice further confirms that the efficacy of AT-312 in attenuating morphine place preference is through its action at the NOP receptor.

We recently showed that AT-312 is devoid of intrinsic motivational effects and does not induce CPP or CPA in mice (32). Similarly, we have shown that other NOP-selective agonists such as AT-202 is also devoid of intrinsic rewarding effects in the CPP paradigm in mice, as is the NOP partial agonist AT-200 (36). N/OFQ (i.c.v.) was also shown to be devoid of intrinsic rewarding effects (37) and even though it decreases basal dopamine levels in the NAc (14), it shows only a mild conditioned place aversion in mice (23). Among other nonpeptide NOP agonists, Ro 65-6570 showed no intrinsic rewarding effects in the CPP paradigm in rats (30); but, the closely related Ro 64-6198 appeared to induce a place preference in rats in this same study, an effect reversed by dopamine D2 receptor antagonist haloperidol but not by a NOP antagonist or opioid antagonist naloxone (30). Taken together, it appears that nonpeptide NOP agonists lack intrinsic rewarding effects and show selective attenuation of the acquisition of morphine CPP through their action at the NOP receptor.

Opioids and psychostimulant drugs are known to produce hyperlocomotion in rodents. Exogenously administered (i.c.v) N/OFQ (1–10 nmol) inhibits spontaneous locomotor activity in mice (12, 38–40) and rats (41). Icv N/OFQ has also been shown to block the locomotor stimulant effect produced by cocaine in rats (20, 21) although N/OFQ had no effect on morphine-induced locomotor sensitization (22). Small-molecule NOP agonists such as Ro 64-6198 (i.p.) also decreased locomotor activity in mice at 1 and 3 mg/kg doses for 30 min after administration, returning to normal at 60 min (42). One concern with compounds that cause motor sedation is that this may have confounding effects in the place conditioning paradigm. AT-312 was also found to decrease motor activity in wild-type mice in this study. However, since the CPP test is conducted in a drug-free state, we believe that the motor-suppressing effect of the drug is not the reason for the inhibitory effect of AT-312 on the place preference. While AT-312 completely blocked acquisition of morphine CPP, it only modestly reduced the motor stimulatory effect of morphine in wild-type mice (Figure 2, upper right panel). This effect was via the NOP receptor because AT-312 did not reduce morphine-induced motor stimulation in NOP knockout mice (Figure 2, lower right panel). On the contrary, in NOP knockout mice, AT-312 pretreatment appeared to enhance the motor stimulatory action of morphine (Figure 2, lower right panel). It is tempting to conclude that this effect of AT-312 on morphine locomotor activity in the absence of the NOP receptor, is due to the low level of MOP partial agonist activity of AT-312 (32). However, this may be less likely because the CPP response was comparable between the vehicle and the AT-312-treated groups in the NOP knockout mice (Figure 1, lower panel). The use of a MOP receptor antagonist in NOP knockout mice should shed more light in this regard. Together, it appears that AT-312 selectively blocks the rewarding and motor stimulatory actions of morphine through the NOP receptor. Furthermore, this effect is not through a non-selective reduction in the motor activity of mice, as we recently demonstrated that other sedative-hypnotic drugs such as pentobarbital, which robustly reduce motor activity in mice, do not diminish the place preference induced by ethanol, even when administered at motor-suppressive doses (32).

N/OFQ (i.c.v.) has been shown to reduce the rewarding effects of cocaine in mice (23). However, studies with small-molecule NOP agonists thus far have shown inconsistent results in their effects in the place conditioning paradigm in rodents (30, 43, 44). For instance, Sartor and colleagues reported that SR-8993, a highly selective nonpeptide agonist failed to block expression, acquisition or reinstatement of cocaine CPP in mice (43). However, the NOP full agonist Ro 65-6570 significantly attenuated cocaine CPP in rats, and the effect was particularly robust when the pretreatment time was increased to 15-min prior to cocaine injections (30). We observed a complete blockade of cocaine-induced CPP in wild-type mice by AT-312 and this response was abolished in mice lacking NOP, suggesting that the inhibitory action of AT-312 was via the NOP receptor. We also found that AT-312 robustly reduced the motor stimulatory effect of cocaine in wild-type mice, which is consistent with our earlier results using N/OFQ (20, 45). Although we do not know why the previous study with small-molecule NOP agonist SR-8993 failed to show an effect on the rewarding action of cocaine, one cannot rule out the impact of pharmacokinetic profiles and pretreatment intervals, which could be a potential confound in terms of their effectiveness in reducing the rewarding effects of cocaine (43). We reported the pharmacokinetic profile and brain penetration of AT-312 in mice (32) and have observed in our studies that AT-312 works within minutes of administration, and that its peak plasma and brain concentration occur within 30 min. Thus, with our pretreatment interval of 5-min and testing period of 30-min, we observe a robust inhibitory effect of AT-312 on cocaine CPP. It is possible that for NOP agonist SR-8993, the reported pretreatment times of 30 min or 2 h before cocaine administration may have missed the window of effect of this compound on cocaine-induced CPP. Further studies with new chemically unrelated selective NOP agonists are certainly warranted to clear the inconsistencies observed thus far with nonpeptide NOP agonists in cocaine reward.

There are different phases of CPP, the acquisition when the CPP response develops and the expression of the CPP response, when the CPP response is measured. In the present study we only assessed the effect of the NOP agonist on the acquisition of the CPP response and tested animals in a drug-free state to rule out the impact of motor impairment on the expression of the CPP response, given that NOP agonists are known to cause motor suppression. However, further studies are needed to assess the impact of NOP agonists on the expression of the CPP response, on the CPP response once it is fully developed and on extinction and reinstatement processes.

NOP agonists have been shown to impact learning and memory processes. In particular, NOP agonists are known to impair several learning tasks and various types of memory (46–53) whereas NOP knockout mice show enhanced memory (50, 54, 55). Therefore, one might argue that AT-312 decreases acquisition of CPP induced by morphine, cocaine (or alcohol in our previous study) by affecting cognitive processes rather than acting on reward-related processes. However, not all types of memory are affected similarly by activation of the NOP receptor. While N/OFQ has been shown to block acquisition of cocaine- as well as morphine-induced CPP, it failed to reduce acquisition of naloxone-induced conditioned place aversion (23). Likewise, N/OFQ has been reported to reduce the expression of morphine-induced CPP at doses that did not alter the expression of naloxone-induced conditioned place aversion (56).

In summary, these results add to our observations that nonpeptide NOP agonists like AT-312 block the rewarding effects of several abused drugs such as morphine, cocaine and alcohol, and may be a promising approach for opioid and psychostimulant addiction pharmacotherapy. Given that addiction to multiple substances is also quite prevalent and there are limited therapeutic options, NOP receptor agonists may have broad therapeutic utility for treating polydrug addiction.

## Author contributions

NZ and KL conceived of the research, supervised the research and wrote the paper. KL analyzed the data. PM and AH conducted the CPP experiments. MM synthesized the test drug.

### Conflict of interest statement

NZ and MM are employees of Astraea Therapeutics. The remaining authors declare that the research was conducted in the absence of any commercial or financial relationships that could be construed as a potential conflict of interest.
